# The impact of family socioeconomic status on adolescent mental and physical health: the mediating role of parental involvement in youth sports

**DOI:** 10.3389/fpubh.2025.1540968

**Published:** 2025-02-26

**Authors:** Wenli Yang, Zubing Xiang, Hong Hu, Haoyuan Zheng, Xin Zhao

**Affiliations:** ^1^School of Physical Education, Chongqing University, Chongqing, China; ^2^Bashu Science City Secondary School, Chongqing, China

**Keywords:** family socioeconomic status, adolescents’ physical health, adolescents’ mental health, parental involvement in youth sports, the mediating role

## Abstract

**Introduction:**

The physical and mental health of adolescents is a crucial cornerstone for social development. Therefore, this study aimed to examine whether family socioeconomic status made a difference in Chinese teenage mental and physical health and to disentangle the mediating role of parental involvement in youth sports in the process in which family socioeconomic status influenced adolescent health.

**Methods:**

A quantitative analysis used a sample of approximately 11,000 adolescents from Chinese middle schools. The research employed structural equation modelling (SEM) to explore the relationships among family socioeconomic status, parental involvement in youth sports, and adolescent mental and physical health.

**Results:**

The findings indicated that both family socioeconomic status and parental involvement in youth sports significantly positively predict levels of adolescents’ physical health and mental health. Further analysis revealed that parental involvement in youth sports mediated the relationship between family socioeconomic status and adolescent health.

**Discussion:**

It is evident that parental involvement in youth sports plays a crucial role in adolescent mental and physical health. Regardless of family socioeconomic status, parents should actively engage in sports activities with their children, which is not only an important way to promote adolescents’ health but also a manifestation of realizing health equity.

## Introduction

Family socioeconomic status (SES) is a significant factor influencing adolescents’ physical and mental health, demonstrating an important relationship with adolescent health (1, 2). As one of the most crucial social determinants of health, low SES is considered the greatest threat to health by the World Health Organization (3). SES is associated with a wide array of health, cognitive, and socioemotional outcomes in children, with effects beginning before birth and continuing into adulthood (2). Adolescents from different family socioeconomic status often exhibit varying characteristics, among which there exist commonalities. Young adults and middle-aged adults in poverty are multi-morbid compared to their wealthier peers. There is a significant negative association between measures of SES and BMI (4). In a meta-analysis, youth with lower socioeconomic status had greater psychopathology and SES was more strongly related to behavior problems than depression or anxiety (5, 6). Parents, as the primary caregivers of most adolescents, play a vital role in their development. Parents from different socioeconomic backgrounds demonstrate varying attitudes toward their children’s lifestyle and education (7). Children’s wellbeing is positively associated with socioeconomic status (8), so low-SES students may experience more threat, more health problems, more negative emotions (9) Parental involvement can make up for parents’ scarcity of economic and social capital and promote children to participate in more sports activities and help improve their overall health (10).

Adolescence is a critical period of rapid physical and psychological development, where parental accompaniment is an important determinant for ensuring adolescents’ mental health (11). Tangible supportive behaviors by parents, including co-participation and transportation, could positively influence PA levels of their offspring (12) and then promote the health-related physical fitness of adolescents (13). Among different types of parental support behaviors, participating in sports activities with children is particularly effective in promoting adolescents’ engagement in physical exercise (12, 14), which in turn enhances their overall health (15–17).

### Socioeconomic status and health

Socioeconomic status (SES) is a comprehensive sociological concept. Mueller and Parcel (18) defined it as the relative position of individuals or families in the social hierarchy, based on the acquisition or control of wealth, prestige, and power. This conceptual framework which laid the foundation for further research guides this paper to conduct an in-depth exploration of how SES shapes individuals’ life trajectories and impacts on people’s well-being. Findings from research have shown that disparities in SES, as a manifestation of social inequality, significantly influence individuals’ health lifestyle choices, leading to profound effects on their health status (19). According to The social causation hypothesis, individual health that is related to socially determined structural factors means that an individual’s position within the social structure determines their health outcomes, for example, those with lower SES typically experience poorer health (20). The impact of SES extends throughout the life cycle, with socioeconomic inequality resulting in differences in health behaviors and physical health status (21). Max Weber’s classical theoretical framework posits that class, status, and power collectively form an individual’s social position (18). Class is primarily determined by economic factors, status corresponds to an individual’s social prestige and represents the degree of social and cultural support one can access, and power is related to political background (22). Weber’s social stratification research provides profound theoretical insights into understanding SES through the three dimensions. These elements interact to delineate an individual’s position within the social structure, thereby influencing their access to health resources and health outcomes. SES is an important reference for measuring an individual’s position, and researchers usually quantify SES using income, occupation, and educational attainment as indicators (2, 4). Due to children’s and adolescents’ absence from the labor market, the SES of their families or parents serves as the primary basis for assessing childhood and adulthood socioeconomic position (23). This underscores the significance of family background about the children and adolescents’ health. For a long time, SES has been viewed as a fundamental factor of disease and health (24, 25). Empirical research consistently confirms the positive influence of family SES on adolescents’ physical health (26) and mental health (27). Adolescents from low SES backgrounds are at a higher risk of depression (28–30) and tend to report poorer self-evaluated health (31, 32).

### Socioeconomic status and parental involvement in youth sports

The specific parenting behavior of “parental involvement in youth sports,” which refers to the frequency with which adolescents participate in sports activities with their parents, is intrinsically linked to family socioeconomic status (SES). According to ecological systems theory, the impact of external forces (such as parental SES) on children is mediated through parenting processes (33). Parental involvement in youth sports, as a kind of practice within the parenting process, is a vital means by which parents fulfil their roles. It allows parents to directly engage in their children’s daily lives and foster parent–child interaction through sports activities. While existing researches broadly address the concept of parental involvement and explore its relationship with SES, studies directly focusing on parental involvement in youth sports remain limited. Most research employs the broader view of parental involvement to analyze social issues related to adolescents.

Currently, there is still some debate over the specific concept of parental involvement. Based on social capital and social closure theories, Coleman (34) systematically categorized parental involvement as family internal involvement and external involvement. This classification has gained widespread application due to its operability in empirical research. Coleman further noted that parental involvement is a comprehensive reflection of family and community social capital, affecting children’s development. The differences in the intergenerational transmission of SES often manifest in the quantity and quality of parental involvement. Parental involvement in youth sports, as a form of internal family participation, directly involves communication and interaction between parents and children. It is also influenced by SES. Parents’ decisions regarding which activities their children participate in and what material and cultural resources are available to them are typically determined by their SES (35, 36). Low SES may limit families’ opportunities to engage in physical activities due to a lack of transportation and financial resources (37). What’s more, the greater levels of cultural capital possessed by parents with high SES can effectively promote their children’s positive development (38–40) and engagement in extracurricular sports activities, as a way to accumulate cultural capital, may facilitate children’s future socioeconomic success (41). Parents with high SES may have a better understanding of the health benefits associated with physical activity (42) and thus they may be more inclined to participate in sports activities with their children.

Furthermore, some researchers have found that family SES can influence parents’ emotions, behaviors and parenting style (7, 43, 44). Parents with low-SES are more likely to have less psychological well-being compared to those from middle or high-SES backgrounds, which significantly predicts a low level of parental emotional warmth (45). This may lead to a lack of parental involvement in youth sports among adolescents from low SES families.

### Parental involvement in youth sports and health

Parental involvement in youth sports is a unique link to the parenting process and its importance becomes more apparent. Multiple studies indicated that parental involvement in youth sports not only positively influences adolescents’ weight management but also has profound effects on their psychological well being. Niemeier et al. (46) conducted a systematic review and meta-analysis revealing that parental participation in weight-related health interventions significantly reduced the body mass index (BMI) of children and adolescents, and then this underscored the notable effectiveness of parental involvement in youth sports for adolescents’ weight control and obesity prevention. Additionally, research by Babkes and Weiss (47) highlighted that parental involvement in youth sports could elicit more positive psychosocial responses in children. The positive correlation between parental involvement in youth sports and adolescents’ levels of physical activity (PA) has also been substantiated (48). Verloigne et al. (49) found in their systematic review that family correlates with adolescents’ energy balance-related behaviors, and parental involvement in youth sports is identified as the most important, positive correlates of physical activity.

High levels of PA have been widely proven to have comprehensive positive impacts on adolescents’ health, including reduced risk of cardiovascular diseases and improved physical fitness (50). Further research by Ghekiere et al. (51) involving 919 Australian children aged 10–12 showed that parental accompaniment when walking or cycling was significantly positively associated with the frequency of children’s walking or cycling trips each week. Furusa et al. (52) found that parents can influence children’s enjoyment of their sporting experience through parents’ active engagement with their child in sports. These findings suggested that parental involvement in youth sports not only directly encouraged children’s participation in physical activities but also laid a solid foundation for long-term health.

In summary, family socioeconomic status is related to adolescents’ physical health and mental health, and also has an important influence on parental involvement in youth sports. Parental involvement in youth sports has a positive impact on improving adolescents’ physical and mental health. Currently, research on parental involvement in youth sports is still limited, and there are few studies that explore the relationship among family socioeconomic status, parental involvement in youth sports, and health together. This study utilizes data from the China Education Panel Survey (CEPS) to examine the relationships among adolescents’ family socioeconomic status, parental involvement in youth sports, and levels of adolescents’ physical health and mental health, aiming to further clarify the role of parental involvement in youth sports in the process by which family socioeconomic status influences adolescent health. Therefore, this study proposes the following hypotheses:

*H1:* Family socioeconomic status has a positive impact on different dimensions of adolescent health.

*H2:* Parental involvement in youth sports is a mediating variable linking family socioeconomic status and adolescent health; family socioeconomic status promotes adolescent health by increasing parental involvement in youth sports.

### Data, variables, and methods

#### Data

This study utilizes data from the baseline survey of the China Education Panel Survey (CEPS) conducted in 2013–2014. The survey was designed and implemented by the National Survey Research Center (NSRC) at Renmin University of China. It is the first large-scale, nationally representative tracking survey project in China that starts from the junior high school stage. In 2013, the CEPS adopted a stratified, multi-stage probability proportional to size (PPS) sampling method. A total of 112 schools and 438 classes were surveyed nationwide, with all students in the selected classes included in the sample. The baseline survey collected data from approximately 20,000 samples. The CEPS database contains relevant information on students’ gender, family socioeconomic status, self-reported physical health, psychological health test items, and parental involvement in physical activities with their children, which are essential for addressing the core questions of this research. After deleting samples with missing values and outliers related to the core variables, a sample of 11,003 students was retained for empirical analysis.

#### Variables

##### The dependent variables

###### Physical health

**Figure 1:**
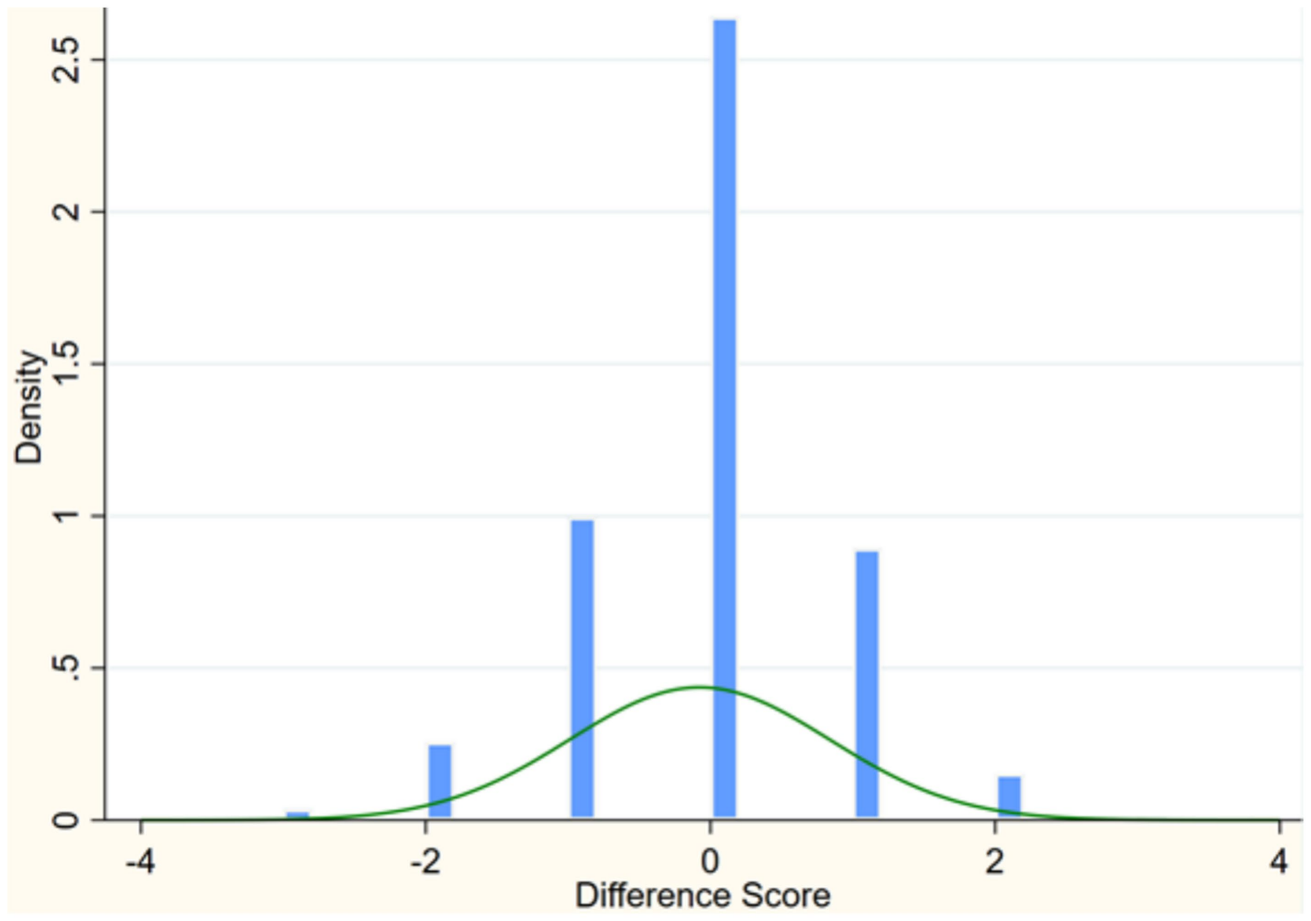
Discrepancies between self-rated health and parent-rated health.

The CEPS questionnaire includes three items to measure adolescents’ physical health: “How would you rate your current overall health? Very poor, Poor, Average, Good, Excellent; How would you rate your child’s current health? Very poor, Poor, Average, Good, Excellent; Compared to your peers, how do you rate your current health? Significantly unhealthy, Less unhealthy, Average, Healthier, Significantly healthy.” The values of 1–5 were assigned to the responses, of which 1 represented the poorest and 5 represented the best. Physical health was measured by the three questions. Lundberg et al. conducted a reliability test for self-evaluated health, validating self-evaluated health against a range of other health issues and then self-evaluated health demonstrated good overall reliability (53). Previous studies suggested that self-evaluated health is a subjective report indicator with high reliability (54, 55). We measured adolescents’ physical health using the question: “How would you rate your current overall health?” Additionally, upon examining the sample data, we identified cases where self-rated health and parent-rated health showed significant discrepancies. To accurately reflect the true health status of the respondents and identify potential outliers in the differences between self-assessment and parental assessment, we calculated the Z-scores of these differences. Among the 12,138 samples, a total of 1,135 observations had Z-scores exceeding ±2 standard deviations and were flagged as outliers, as illustrated in Figure 1. Sensitivity analysis confirmed that excluding these outliers did not significantly alter our main results.

###### Mental health

The CEPS questionnaire set mental health scale, consisting of five items: “In the past seven days, did you have the following feelings: sadness, depression, unhappiness, a sense of life being meaningless and grief?” The values of 1–5 were assigned to “Always, Often, Sometimes, Seldom and Never” respectively. Mental health was measured by the five questions. The reliability coefficient (a) for the mental health scale was 0.85, and it has been employed in studies regarding the mental health of Chinese adolescents (56). The summary description of the dependent variables is provided in Table 1.

**Table 1:** The descriptive statistics of the dependent variables.

Latent variable	Observed variable	Variable coding	Frequency	Percentage
Mental health	A1: in the past seven days, did you have the following feelings: sadness?	5 “Never”	252	2.29%
		4 “Seldom”	512	4.65%
		3 “Sometimes”	3,679	33.44%
		2 “Often”	3,799	34.53%
		1 “Always”	2,761	25.09%
	A2: in the past seven days, did you have the following feelings: depression?	5 “Never”	250	2.27%
		4 “Seldom”	608	5.53%
		3 “Sometimes”	2,275	20.68%
		2 “Often”	3,344	30.39%
		1 “Always”	4,526	41.13%
	A3: in the past seven days, did you have the following feelings: unhappiness?	5 “Never”	336	3.05%
		4 “Seldom”	762	6.93%
		3 “Sometimes”	3,376	30.68%
		2 “Often”	3,867	35.15%
		1 “Always”	2,662	24.19%
	A4: in the past seven days, did you have the following feelings: a sense of life being meaningless?	5 “Never”	332	3.02%
		4 “Seldom”	440	4.00%
		3 “Sometimes”	1,432	13.01%
		2 “Often”	2,483	22.57%
		1 “Always”	6,316	57.40%
	A5: in the past seven days, did you have the following feelings: grief?	5 “Never”	303	2.75%
		4 “Seldom”	483	4.39%
		3 “Sometimes”	2,448	22.25%
		2 “Often”	3,814	34.66%
		1 “Always”	3,955	35.95%
Physical health	How would you rate your current overall health? (Self-rated health)	1 “Very poor”	10	0.09%
		2 “Poor”	287	2.61%
		3 “Average”	2,157	19.60%
		4 “Good”	4,354	39.57%
		5 “Excellent”	4,195	38.13%
	How would you rate your child’s current health? (Parent-rated health)	1 “Very poor”	13	0.12%
		2 “Poor”	209	1.90%
		3 “Average”	2,193	19.93%
		4 “Good”	4,250	38.63%
		5 “Excellent”	4,338	39.42%
	Compared to your peers, how do you rate your current health?	1 “Significantly unhealthy”	133	1.22%
		2 “Less unhealthy”	533	4.84%
		3 “Average”	3,078	27.97%
		4 “Healthier”	4,438	40.33%
		5 “Significantly healthy”	2,821	25.64%

##### Independent variables

The independent variable, Family Socioeconomic Status, was a latent variable in this study, typically measured by indicators such as parental occupation, education level, and family income (57). However, some researchers argued that measuring family income was difficult in the context of Chinese households and suggested that it can be omitted, as occupation and education level adequately reflected a family’s socioeconomic status (58). Furthermore, the CEPS database lacked objective and direct information regarding family economic conditions (e.g., annual income, annual expenditure). The CEPS questionnaire included questions related to parental education level and occupation, specifically: “What does your mother do for work?” and “What does your father do for work?” as well as the educational levels of the student’s father and mother. Therefore, this study used the father’s occupation, mother’s occupation, father’s education level, and mother’s education level as observed variables to measure family socioeconomic status. The basic description of the independent variables was presented in Table 2.

**Table 2:** The descriptive statistics of the independent variables and mediating variables.

Latent variable/Mediating variables	Observed variable	Variable coding	Frequency	Percentage
Latent variable: family socioeconomic status	Father’s education level	1 “Elementary school and below”	1,538	13.98%
		2 “Junior high school”	5,427	49.32%
		3 “Senior high school”	2,238	20.34%
		4 “Bachelor”	1,656	15.05%
		5 “Master”	144	1.31%
	Mother’s education level	1 “Elementary school and below”	2,529	22.98%
		2 “Junior high school”	5,206	47.31%
		3 “Senior high school”	1,756	15.96%
		4 “Bachelor”	1,409	12.81%
		5 “Master”	103	0.94%
	Father’s occupation	1 “Unemployment”	286	2.60%
		2 “Manual labor”	8,538	77.60%
		3 “Mental labor”	2,179	19.80%
	Mother’s occupation	1 “Unemployment”	1,066	9.69%
		2 “Manual labor”	8,275	75.21%
		3 “Mental labor”	1,662	15.10%
Mediating variables: parental involvement in youth sports		1 “At least once a week”	6,549	59.52%
		2 “Less than once a week”	4,454	40.48%

### Occupation

The occupation variable was reclassified and reassigned based on existing research, categorizing occupations as follows: (1) Unemployment: including joblessness and layoff; (2) Manual labor: including farmers, skilled workers, drivers, general employees, and self-employed individuals; (3) Mental labor: including leaders and staff of government agencies and public institutions, senior and mid-level leaders in enterprises/companies, teachers, engineers, doctors, and lawyers, with values assigned sequentially from 1 to 3.

### Education level

Based on their academic qualifications, parents’ education levels were categorized into five levels: elementary school and below, junior high school, senior high school, bachelor, master and above, with values assigned sequentially from 1 to 5.

#### Mediating variables

Parental Involvement in Youth Sports was measured by the question: “Frequency of doing the following activities with your parents – exercising.” The responses included six options: “1 = never”; “2 = once a year”; “3 = once every six months”; “4 = once a month”; “5 = once a week”; “6 = more than once a week.” In this study, the options “once a week” and “more than once a week” were combined and revalued as “at least once a week” (assigned a value of 2), while the other options are combined and revalued as “less than once a week” (assigned a value of 1). The basic description of the control variables was presented in Table 2.

## Methods

**Figure 2:**
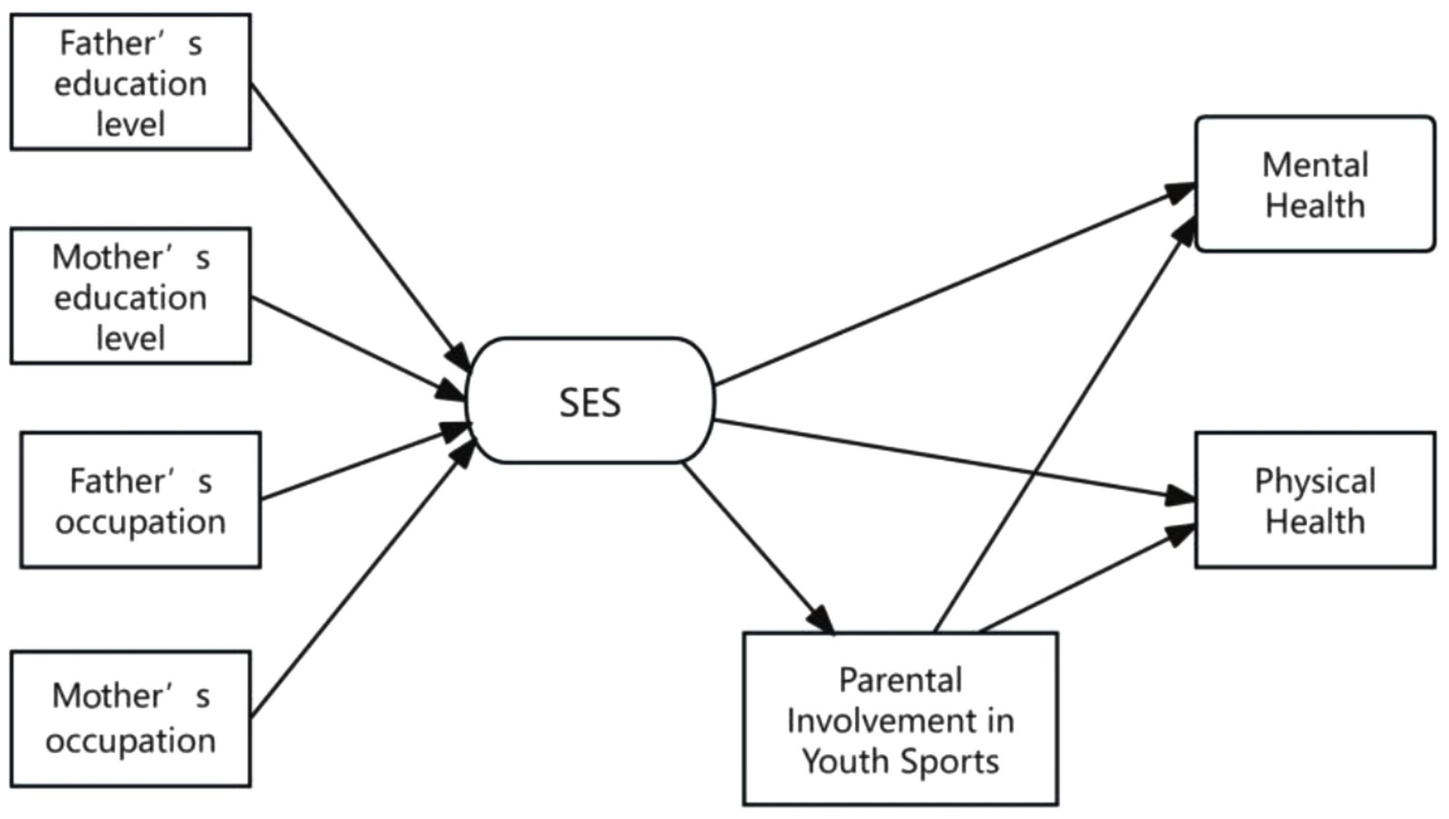
Theoretical model. The arrows represent the impact paths.

To validate the research hypotheses proposed in this study, we employed structural equation modeling (SEM) using Stata 16.0 statistical software. SEM is a statistical method for exploring the relationships and structures between theories and concepts, integrating ideas and techniques from factor analysis, path analysis, and multiple linear regression analysis. This study utilized two sets of structural equation models: the baseline model and the mediation model. First, the baseline model established a direct path effect between family socioeconomic status and adolescent health to test Hypothesis 1. Second, the mediation model added the mediating variable of parental involvement in youth sports to the baseline model, examining the path effects among family socioeconomic status, parental involvement in youth sports, and adolescent health to test Hypothesis 2. The theoretical model for this study was constructed based on existing literature and the research hypotheses. The theoretical framework of the study was illustrated in Figure 2.

Additionally, a model deemed satisfactory should meet a set of criteria. A standardized root mean square residual (SRMR) value of 0.000 indicates a perfect model-data fit, while a value below 0.08 was considered acceptable. Regarding parsimony correction indices, the root meant square error of approximation (RMSEA) is viewed as an “approximate error” index. An RMSEA estimate and its corresponding 90% confidence interval below 0.10 suggested a good model-data fit. For comparative fit indices, the comparative fit index (CFI) ranged from 0.000 to 1.000, with values closer to 1.000 indicating a better model-data fit (59).

## Results and analysis

### The impact of family socioeconomic status on adolescent health

Since the ML estimator is unbiased and consistent under large sample conditions and was the most commonly used estimation method in structural equation modeling, this study employed the maximum likelihood estimation method for model testing. The measurement models of the latent variables SES and physical health demonstrated good reliability and validity (CR for mental health = 0.855 > 0.7, AVE = 0.543 > 0.5, √AVE = 0.736 > 0.05; CR for SES = 0.716 > 0.7, AVE = 0.450, √AVE = 0.670 > 0.153. Although the AVE value for SES was slightly below the ideal threshold of 0.5, previous research had emphasized that all four indicators were crucial for measuring family socioeconomic status. This discrepancy may be attributed to the absence of a key variable for family socioeconomic status in the database. However, the composite reliability (CR) and discriminant validity (√AVE) of SES met the required thresholds. Therefore, despite the slightly lower AVE, we considered the results acceptable.) The fit indices, including RMSEA and CFI, for the baseline model were presented in Table 3. The baseline model analysis indicated that family socioeconomic status significantly affected adolescent health, with unstandardized path coefficients of 0.407 for mental health and 0.274 for physical health, both positive and statistically significant at the *p* < 0.01 level. These findings confirmed that family socioeconomic status positively influenced adolescent health, with the strongest effect on physical health, supporting Hypothesis 1. Furthermore, within the measurement model of family socioeconomic status, mother’s education level (0.798) exhibited the highest factor loading, followed by father’s education level (0.793). The factor loading for father’s occupation is 0.549, while mother’s occupation had the lowest factor loading (0.479).

**Table 3:** The structural equation modeling testing of the relationship between family socioeconomic status and adolescent health.

Effects	Mental health	Physical health
Family socioeconomic status	0.274^***^	0.407^***^
RMSEA	0.076	
CFI	0.939	
SRMR	0.073	
TLI	0.919	

***p < 1%; **p < 5%; *p < 10%.

### The mediating role of parental involvement in youth sports

To explore the mediating effects of parental involvement in youth sports, the mediating variable “parental involvement in youth sports” was added to the baseline model, resulting in the establishment of a mediating model. We again employed the maximum likelihood estimation method for testing, and the analysis results of the mediating model were presented in Table 4. In the mediating model, family socioeconomic status significantly impacted both parental involvement in youth sports and adolescent health and the unstandardized path coefficients for parental involvement in youth sports, mental health and physical health are all positive and significant at the *p* < 0.01 level. Furthermore, the unstandardized path coefficients for the mediating variable parental involvement in youth sports with mental health and physical health were also positive and significant at the same level. Notably, the unstandardized path coefficient between parental involvement in youth sports and adolescent mental health was higher than that for physical health.

**Table 4:** The structural equation modeling testing of the mediating role of parental involvement in youth sports.

Effects	Direct effects	Indirect effects	Overall effects
Family socioeconomic status- > Mental health	0.151^***^	0.116^***^	0.266^***^
Family socioeconomic status- > Physical health	0.304^***^	0.094^***^	0.398^***^
Family socioeconomic status- > Parental involvement in youth sports	0.472^***^		0.472^***^
Parental involvement in youth sports- > Mental health	0.246^***^		0.246^***^
Parental involvement in youth sports- > Physical health	0.199^***^		0.199^***^
RMSEA	0.068		
CFI	0.942		
SRMR	0.064		
TLI	0.923		

***indicates significance at the 1% level, **at the 5% level, and *at the 10% level.

Thus, the mediating model revealed that parental involvement in youth sports played an important role in the process in which family socioeconomic status affected adolescent health. Family socioeconomic status positively influenced parental involvement in youth sports, which in turn positively impacted adolescent health. Therefore, family socioeconomic status indirectly influenced adolescent health by affecting parental involvement in youth sports, confirming Hypothesis 2.

**Figure 3:**
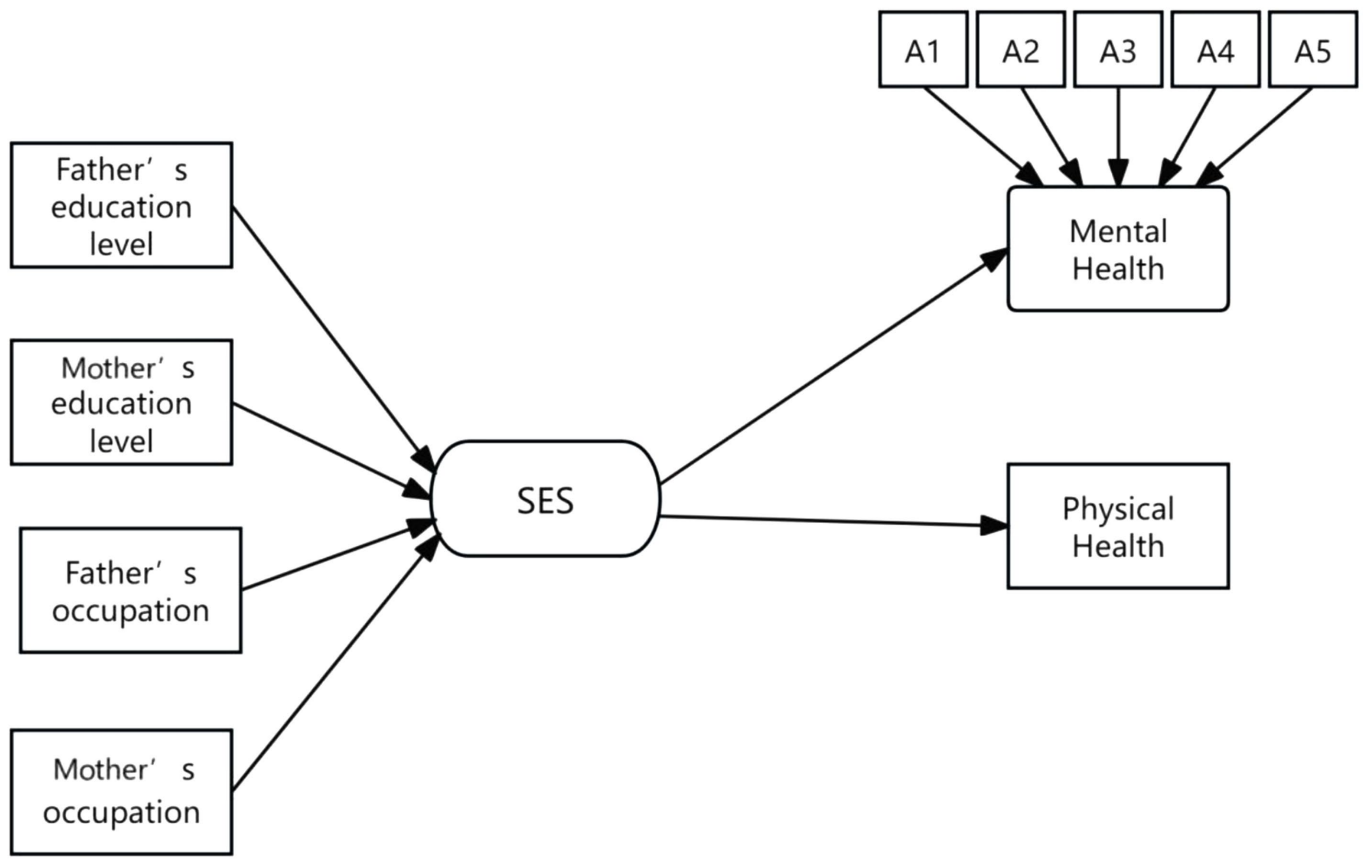
The path of the baseline model. The arrows represent the impact paths; A1 represents: in the past seven days, did you have the following feelings: sadness?; A2: in the past seven days, did you have the following feelings: depression?; A3: in the past seven days, did you have the following feelings: unhappiness?; A4: in the past seven days, did you have the following feelings: a sense of life being meaningless?; A5: in the past seven days, did you have the following feelings: grief?

**Figure 4:**
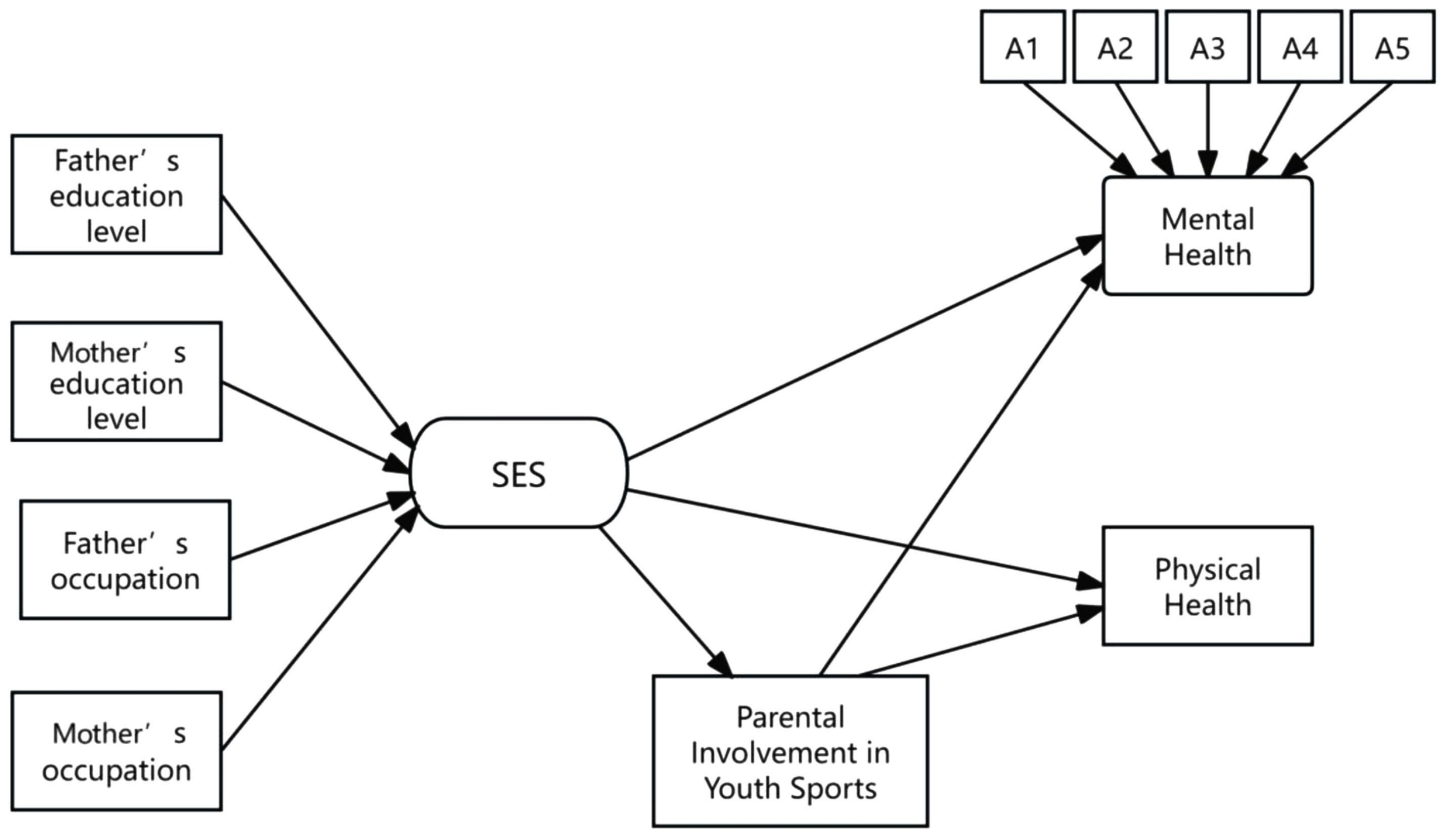
The SEM of the mediating role of parental involvement in youth sports. The arrows represent the impact paths; A1 represents: in the past seven days, did you have the following feelings: sadness?; A2: in the past seven days, did you have the following feelings: depression?; A3: in the past seven days, did you have the following feelings: unhappiness?; A4: in the past seven days, did you have the following feelings: a sense of life being meaningless?; A5: in the past seven days, did you have the following feelings: grief?

To better illustrate the impact of the mediating variable, Figures 3, 4 presented the paths of the baseline model and the mediating model, respectively. The examination results indicated that multiple fit indices for both models generally met the standards, achieving acceptable levels of fit.

Based on the path models in Figures 3, 4, two main findings can be summarized. First, the data analysis confirmed the significant role of socioeconomic status (SES) in adolescent physical and mental health. Specifically, as family SES increases, adolescents tended to exhibit better psychological and physical health. Second, the study provided empirical evidence for the mediating role of parental involvement in youth sports. More specifically, parental involvement in youth sports served as a crucial mediating variable between family SES and adolescent physical and mental health, indicating that SES influences adolescent health partly through the frequency of parental involvement in youth sports. The data further showed that higher SES families tended to have more frequent parental involvement in youth sports, and compared to adolescents who lack such companionship, those whose parents accompanied them in sports activities at least once a week experienced significant improvements in both physical and mental health. It plays a particularly important role in adolescent mental health.

## Discussion

Through the exploration of family socioeconomic status, adolescent health, and parental involvement in youth sports, we found that family socioeconomic status could positively predict the health levels of adolescents and adolescents from families with high socioeconomic status showed significantly better health compared to those from low socioeconomic status families. This finding aligned with numerous existing studies that there is a critical role of family socioeconomic status during adolescence (4, 6, 60). Research has shown that the family sports environment is a key determinant of adolescents’ regular participation in sports (39, 61). Adolescents are less likely to engage in physical activities if their parents are inactive (12, 62). Further research indicated that parents from high-SES families typically had a stronger awareness of health (63) and greater flexibility in both time and finances (64), making them more likely to engage in physical activities with their children.

The findings highlighted the crucial mediating role of parental involvement in youth sports between socioeconomic status and adolescent health. Adolescents in China rarely engaged in sports activities with their parents, particularly those from low socioeconomic backgrounds, where parental participation was notably low. Our study introduced the hypothesis that parental involvement in youth sports acted as a mediating variable and empirically tested this hypothesis. This aligned with prior literature and filled a gap, particularly regarding the role of parental involvement in youth sports in mitigating health disparities among adolescents from low-SES backgrounds. Heradstveit (65) suggested that several indicators of parental socioeconomic status, including family economic welfare and parental education levels, were related to a lack of physical activity among adolescents. Previous research has primarily focused on the role of economic resources (10, 37), whereas this study revealed the mediating role of the sociocultural attributes of parental involvement in youth sports. Therefore, encouraging a supportive family sports environment is essential, especially for parents from low socioeconomic backgrounds, to enhance adolescent health.

The influence of SES on adolescent sports participation shows consistency across countries and cultures. In developed countries such as the United States, Norway, and Northern Europe, adolescents from high-SES families are more inclined to engage in organized sports (66, 67). For example, a research from Germany indicated that Children and adolescents with a high SES spent much more time on PA in organized sports (155 min) than children with a low SES (99 min) and low parental SES correlates with more access for children and adolescents to participation in unorganized sports (37). Generally, participation in organized sports is associated with positive developmental outcomes (67–69). Parents are significant others influencing adolescents’ participation in sports. A Danish longitudinal study demonstrated that parental involvement in children’s sport increased the likelihood that the child participated in organized sports, categorizing parents into four types: unengaged, servicing, self-realization, and super parents (70). However, the study also found that involvement from disadvantaged parents boosted children’s participation in organized sports, whereas involvement from advantaged parents had the opposite effect (70). Research from Hungary indicated that there was a positive interrelation between parental involvement in youth sports and sports performance of their children (71). The findings of this study are not only applicable to China but may also be relevant to other developed countries and middle-income countries.

Additionally, economic development is often associated with higher incomes, better education, and improved healthcare, allowing high-SES families to offer their children superior resources in areas such as nutrition, medical care, sports, and education. However, substantial socioeconomic disparities persist across urban and rural areas, regions, and different income groups, leading to significant heterogeneity in the impact of SES on health (72, 73). Parental involvement in youth sports not only directly benefits adolescent health but also helps reduce health disparities caused by SES, providing valuable insights for policies aimed at improving adolescent health.

Despite the findings indicating the mediating role of parental involvement in youth sports, the study did not delve deeper into the differences among various social groups, such as urban versus rural populations, regional variations, and gender differences, due to space limitations. These issues could constitute a relatively independent study requiring detailed theoretical exploration and data analysis. Furthermore, parental involvement in youth sports is an important yet under-researched concept that merits further investigation by scholars. Given the diverse ways of engaging in sports, parental involvement is not limited to traditional activities. With the rapid development of the global economy, sports are increasingly capturing market share and attracting more people to join in sports consumption and physical activities. Younger generations master more kinds of sports than their parents, raising questions about how these new parents will accompany their children in sports activities, which forms of parental sports involvement will be more appealing to children, and how effective these forms will be for health promotion, and parents from different countries and regions are inclined to which forms of parental sports involvement. Future research could analyze parental involvement in youth sports by using samples from various countries, further enriching the theoretical understanding of this topic.

### Recommendations

#### Focus on children from low socioeconomic status families: implement early education intervention policies to bridge the gap

Addressing the developmental disparities of children from low socioeconomic status families is crucial for promoting health equity and educational balance in China. Policymakers should recognize the underlying mechanisms through which family socioeconomic status influences children’s academic outcomes and consider compensatory policies for children from low socioeconomic backgrounds. Providing educational resources to children from low socioeconomic families can help mitigate the effects of class stratification. Existing research indicated that compared to parents from high socioeconomic backgrounds, parents with low socioeconomic status demonstrated higher effectiveness in involving in their children’s school education (74). In addition to ensuring fairness in traditional educational systems, current inequalities in family education also need attention. The important role of parental involvement in youth sports highlights potential health risk factors for adolescents.

#### Enhance the quality of parental involvement and provide support for effective parental involvement

Parents from different socioeconomic backgrounds should adopt varying strategies for sports companionship. Parents from lower socioeconomic backgrounds should particularly pay more attention to developing skills for parent–child interaction to enhance the quality of their participation in youth sports. The study’s findings reveal ed. that parents from high socioeconomic backgrounds generally have a higher frequency of parental involvement in youth sports than those from low backgrounds. This underscores the need for parents at all socioeconomic levels to prioritize spending time and energy on strengthening communication with their children. Additionally, parents should seek to improve their own sports skills and communication abilities, thereby enhancing the effects of parent–child interactions.

## Conclusion

This study, based on CEPS data and employing Stata 16.0 software, used structural equation modeling to examine the intrinsic connections between family socioeconomic status, parental involvement in youth sports, and adolescent health. The results demonstrated that family socioeconomic status had a positive influence on both adolescent physical and mental health, and the frequency of parental involvement in youth sports, it plays a particularly important role in adolescent mental health. Specifically, family socioeconomic status indirectly affected adolescent physical and mental health by influencing the frequency of parental involvement in youth sports, confirming the existence of a mediating effect. Effective parental involvement in youth sports plays a positive role in adolescent physical and mental health. In summary, families with high socioeconomic status were more likely to actively engage in sports activities with their children, leading to improved health outcomes for the children.

## Data Availability

Publicly available datasets were analyzed in this study. This data can be found at: http://ceps.ruc.edu.cn/.
